# New insights into GluT1 mechanics during glucose transfer

**DOI:** 10.1038/s41598-018-37367-z

**Published:** 2019-01-30

**Authors:** Tatiana Galochkina, Matthieu Ng Fuk Chong, Lylia Challali, Sonia Abbar, Catherine Etchebest

**Affiliations:** Université Sorbonne Paris Cité, Université Paris Diderot, Inserm, INTS, Unité Biologie Intégrée du Globule Rouge UMR S1134, DSIMB, Laboratoire d’Excellence GR-Ex, Paris, 75739 France

## Abstract

Glucose plays a crucial role in the mammalian cell metabolism. In the erythrocytes and endothelial cells of the blood-brain barrier, glucose uptake is mediated by the glucose transporter type 1 (GluT1). GluT1 deficiency or mutations cause severe physiological disorders. GluT1 is also an important target in cancer therapy as it is overexpressed in tumor cells. Previous studies have suggested that GluT1 mediates solute transfer through a cycle of conformational changes. However, the corresponding 3D structures adopted by the transporter during the transfer process remain elusive. In the present work, we first elucidate the whole conformational landscape of GluT1 in the absence of glucose, using long molecular dynamics simulations and show that the transitions can be accomplished through thermal fluctuations. Importantly, we highlight a strong coupling between intracellular and extracellular domains of the protein that contributes to the transmembrane helices reorientation during the transition. The conformations adopted during the simulations differ from the known 3D bacterial homologs structures resolved in similar states. In holo state simulations, we find that glucose transits along the pathway through significant rotational motions, while maintaining hydrogen bonds with the protein. These persistent motions affect side chains orientation, which impacts protein mechanics and allows glucose progression.

## Introduction

Glucose is an essential source of energy for the mammalian cells. Its transport to the cell occurs as the result of the facilitative diffusion governed by the membrane proteins. Human glucose transporter GluT1 is the most rigorously characterized human solute transporter. It is primarily responsible for the cellular uptake of glucose into erythrocytes and endothelial cells of the blood-brain barrier. GluT1 deficiency or inactivating mutations lead to the central nervous system dysfunction characterized by the appearance of drug-resistant epileptic seizures, psychomotor retardation and a delayed development of the cranial box associated with the De Vivo disease^1^. At the same time, over-expression of GluT1 and other glucose transporters is observed for a wide variety of malignant cells^2^.

Glucose transporters (GluTs) belong to one of the largest families of the membrane transporters, Major Facilitator Superfamily (MFS, TCDB: 2.A.1)^3^ present in all the kingdoms of life. More precisely, GluTs make part of the Sugar Porters (SP) sub-family whose members are responsible for the uptake of glucose and other monosaccharides or disaccharides^4^.

All the MFS proteins share a common fold: 12 transmembrane (TM) helices are organized in two distinct domains called N- and C-domains, each consisting of six consecutive helices. Located against each other taking the form of a “V”, these domains form a large hydrophilic cavity at the center of the protein. The amino acids located inside the cavity ensure the ligand binding and thus determine the substrate specificity of the transporter^5^. Opening of the protein cavity to the cytoplasm or periplasm/extracellular medium allows the solute transfer by the MFS proteins. According to the model of the alternating access mechanism^6^, the cycle of the conformational changes of the MFS proteins during the ligand transport includes the outward facing (open to the extracellular medium) conformation necessary for the ligand uptake, the transitional closed states with the ligand located in the protein cavity isolated from the both extracellular and intracellular media, and the inward facing (open to cytoplasm) state allowing the ligand release.

The MFS transport mechanism was supported by experimental studies. Among the resolved X-ray structures of the MFS members, both outward facing, inward facing and closed states were detected for different proteins (Table S1). However, resolution of the same protein in several conformational states remains a major challenge for crystallographers. The only MFS transporter crystallized in three different conformations is the bacterial homolog of GluTs, XylE crystallized in the open to the extracellular medium, partially open to the cytoplasm, and completely open to the cytoplasm conformations^7–9^. Among GluTs, only GluT5 was recently obtained in two distinct states (open to the cytoplasm and open to the extracellular medium for the bovine and murine isoforms respectively)^10^. Human GluT3 was crystallized in several outward facing states^11^, while human GluT1 was obtained only in the inward facing conformation^12,13^.

The first reported X-ray structure of GluT1 was obtained at 3.2 Å resolution for the GluT1 double mutant in the presence of a detergent molecule *n*-nonyl-*β*-D-glucopyranoside (*β*-NG) located in the central cavity (PDB ID: 4PYP^12^). Deng *et al*. substituted the previously identified glycosylation site N45^14^ by threonine in order to prevent potential heterogeneity of the protein structures and added the point mutation E329Q to lock the transporter in the inward facing conformation^15,16^. In 2015 Kapoor *et al*. obtained three wild type GluT1 structures at 3 Å resolution using as ligands two GluT1 inhibitors and Cytochalastin B^13^ (PDB ID: 5EQC, 5EQH and 5EQI respectively). Interestingly, all the three structures exhibit identical conformations with the N45T/E329Q mutant and the inhibitors occupy the same binding site as the *β*-NG sugar moiety^17^.

**Figure 1:**
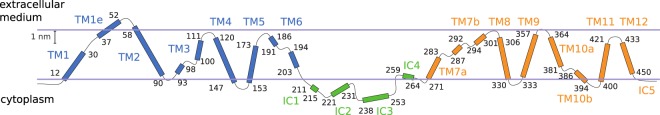
The general Glut1 topology in the membrane.

The three-dimensional structure of GluT1 contains 12 transmembrane (TM) helices characteristic for the MFS fold and also an intracellular domain composed of four *α*-helices (IC helices), which it shares with its bacterial homologs of the SP family, namely XylE and the glucose/H+ symporter (GlcP)^18^, but not with the other MFS transporters with known structures (Fig. 1). The protein C-terminus (IC5 in Fig. 1) is not present in any of the resolved GluT1 structures, probably due to its high flexibility.

The obtained GluT1 structure inspired the development of the molecular models aiming to elucidate the different aspects of the GluT1 solute transfer^19–22^. The glucose pathway through the transporter was predicted using the combination of targeted dynamics to reproduce the protein conformational transition and steered dynamics inducing translocation of the solute^19^. These results were further refined by Fu *et al*.^20^ Four glucose binding sites were identified using ligand docking to the homology model of GluT1 outward facing state and to GluT1 X-ray inward open structure followed by 60 ns molecular dynamics (MD) simulations equilibrating the protein structure for each position of glucose. In both studies^19,20^ the outward facing GluT1 conformation was modelled by homology using as a template GluTs bacterial homolog XylE (PDB ID: 4GBZ). Unfortunately, XylE shares only 29% of the sequence identity and about 49% of the sequence similarity with GluTs^8^ and thus its three-dimensional structure can serve only as an approximation of the GluT1 outward facing state. The GluT1 inward facing conformation during glucose transport can also differ from the available X-ray structures obtained in the presence of the detergents with an aliphatic tail.

In the current work we analyse the GluT1 transitions between the different conformational states in the absence and presence of the solute using conventional MD simulations. In contrast to previous studies^19,20^, we identify the outward facing GluT1 structure by performing long MD simulations of GluT1 WT in the membrane environment starting from its inward facing X-ray structure. According to our simulations, the protein can undergo transition to the outward facing state due to the thermal fluctuations. The obtained conformation demonstrates significantly lower degree of opening than the XylE outward open X-ray structure. We perform a detailed analysis of the corresponding transitions at the level of TM helices as well of the intra- and extracellular parts of the protein. Finally, we use the obtained in MD outward facing GluT1 structure to model the glucose transport by the protein and to analyse the impact of the presence of the solute on the protein conformational mobility.

## Results

To better identify the conformational transitions associated with the MFS proteins transport cycle, we have first focused on the available X-ray structures of the MFS proteins from the SP family (Table S1).

### Location of GluT1 structure in the MFS conformational space

**Figure 2:**
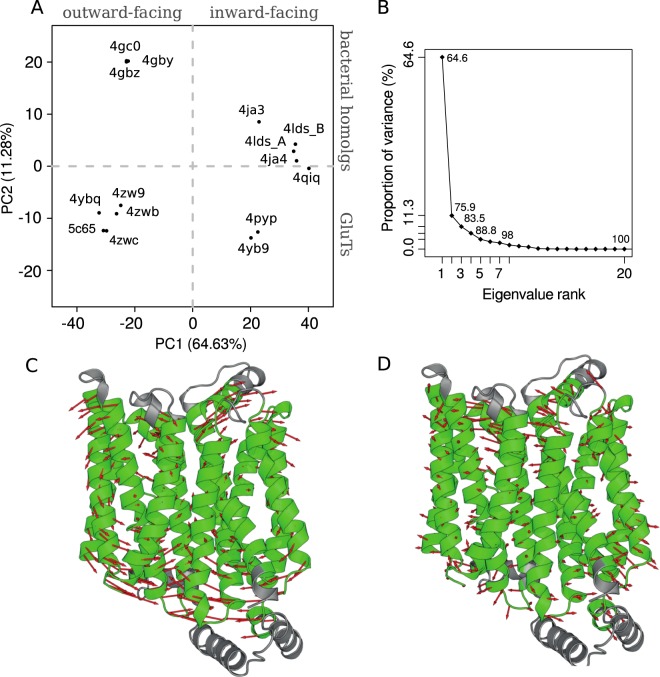
PCA results for the common core of the available MFS X-ray structures: projection of the crystal structures on the subspace formed by the first two PCs (**A**); variance proportion for different eigenvalues obtained in PCA (**B**) and the PC1 and PC2 representation in the vector field of the GluT1 structure (**C**,**D**). The common core is colored in green, the red arrows indicate the movements of C_*α*_ atoms between two extreme values of PC1 and PC2. Projections of all the inward open structures of Glut1 WT (PDB ID: 5EQG, 5EQH, 5EQI) coincide with that of 4PYP.

We have performed structural alignment of all the available X-ray structures (Table S1) and identified their common core part consisting of 339 residues (Fig. S3). The common core covers most of the transmembrane part of the protein and thus includes the large part of the transporter responsible for the conformational transition. We have superimposed the 3D core structures of all the considered homologs and performed a principal component analysis (PCA) of the ensemble (Fig. 2) consisting in the computation and diagonalization of the covariance matrix of the coordinates.

In total, the first three principal components (PCs) constitute 83.5% of the variance, while the contributions of the individual components of higher range fall below 5% (Fig. 2B). The first principal component (PC1) strongly dominates the overall variance distinctly separating the inward facing conformations (Fig. 2A, right half-plane) from the outward facing ones (Fig. 2A, left half-plane). The corresponding transitional movements include global rotation of the N- and C-domains accompanied by the large amplitude displacement of TM helices ends (Fig. 2C), which is in accordance with the analysis of the available X-ray structures of the MFS proteins^4^.

The movements described by the second principal component (PC2) involve mainly the extracellular ends of the helices (Fig. 2D). Such transitions allow us to distinguish the GluT structures from those of the other MFS proteins. Indeed, the GluT conformers appear to be distinctly separated from their bacterial homologs by the values of PC2 (Fig. 2A).

The results of PCA for the X-ray structures of the MFS homologs allowed us to perform an efficient analysis of the GluT1 conformations adopted during MD simulations.

### GluT1 transition from IF to OF state through thermal fluctuations

We have performed a series of 1 *μ*s MD simulations for the GluT1 protein model in the POPC bilayer starting from the inward facing conformation reconstructed from the 4PYP X-ray structure (Fig. S1). During our simulations the protein adopted a range of conformations that we classify as inward and outward facing in case the protein cavity was in contact with the intracellular or extracellular medium respectively, or as closed conformations in case the protein cavity was isolated from both media by the hydrophobic barriers.

**Figure 3:**
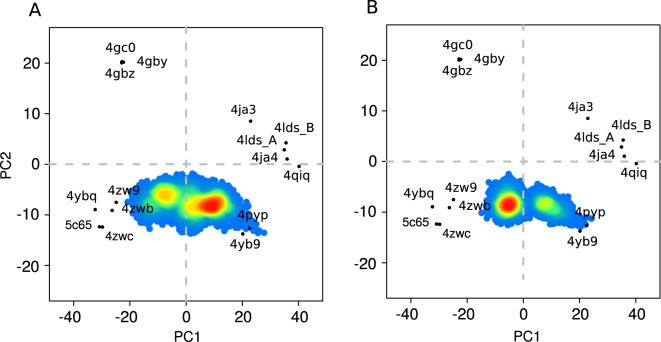
Projection of the conformations adopted by GLUT1 during the set of MD simulations in the absence (**A**) and presence (**B**) of glucose on the plane formed by the first two PCs of the X-ray MFS protein structures (Fig. 2). The color indicates the population density of the corresponding area of the conformational space (increasing from blue to red).

For all the MD simulations we observed a transition of the inward facing conformation to the closed states. For several MD simulations this transition was followed by the opening of the protein cavity to the extracellular side. The major role in such transition was played by the mobility of the intracellular part of the protein demonstrating high RMSF values (Fig. S2). In particular, during the simulation we observed IC5 coming into contact with the other IC helices and thus locking the outward facing conformation from the intracellular side (Fig. S1C). Less prominent conformational changes concern TM part of the protein (Fig. S1A). To analyse the observed conformational transitions at the level of the core part rearrangement, we have projected the GluT1 conformers obtained in the simulations on the subspace formed by the first two PCs determined for the X-ray structures of the MFS members (Fig. 3A).

The area of the conformational space corresponding to the initial inward facing protein conformation is very low populated (Fig. 3A, PC1 equal ~20). At the same time, we observe high population of the closed states (PC1 in range of (−15, 10), Fig. 3A). Among such conformations, we detect several different highly populated areas (Fig. 3A, PC1 lying in the intervals (−16, −14), (−15, −5), (0, 5), (8, 15)). The different closed sates were predicted to make part of the MFS transport cycle and in the presence of ligand are classified as inward facing and outward facing occluded states^4,17^. It is important to note that in our ligand-free simulations we observe the interchange between the different closed states, which suggests that such transitions during the solute transport can appear as the result of the simple thermal fluctuations of the atoms of the transmembrane part of the protein.

**Figure 4:**
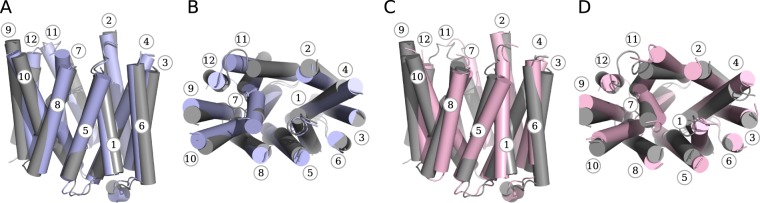
Superposition of the common cores for the outward facing state obtained in MD simulations (gray) with the crystal GluT3 structure 4ZW9 ((**A**,**B**) blue) and with the XylE structure 4GBZ ((**C**,**D**) rose) for the side (**A**,**C**) and top (**B**,**D**) views. Numbers of the TM helices are given in cirlces.

During MD simulations we have also detected several outward facing GluT1 conformers (Fig. 3A, PC1 equal ~−20). The obtained structures appear to be very close to the outward facing occluded X-ray structure of GluT3 4ZW9 (Fig. 4A,B) with RMSD value for the C_*α*_ atoms of the core equal ~2.1 Å. The main difference between two structures concerns the arrangement of the extracellular part of TM7 (Fig. 4B) which was reported to be an important indicator of the GluT3 transition from the occluded to the outward facing state^11^. In our simulations TM7 retains the broken conformation, which leads to the observed difference between our model and the outward facing X-ray GluT3 structure (PDB ID: 4ZWC) in terms of the projection on the (PC1,PC2) plane (Fig. 3A).

Interestingly, the projection of the core structure of the GluT1 bacterial homolog XylE (PDB ID: 4GBZ) has a value of PC1 very close to that of the GluT3 4ZW9 projection (Fig. 2A). Nevertheless, the arrangement of the core differs significantly for the XylE and GluT1 outward facing structures (Fig. 4C,D). The 4GBZ structure demonstrates the higher degree of opening towards the extracellular medium. Indeed, the size of the protein cavity identified on the extracellular side of the GluT1 conformers obtained in the simulation is significantly smaller than that of the X-ray structure 4GBZ (Fig. S4). This effect is especially pronounced for the helices TM5, TM8 and TM11 whose extracellular ends are located further from the center of the protein as compared to the GluT1 structure (Fig. 4D). The second important difference concerns the extracellular part of TM1, which is broken into two parts in all the available GluT1-5 crystal structures but retains an almost straight conformation in XylE (Fig. 4D). As the result, the projection of the outward facing conformations adopted by GluT1 during the MD simulations appear to be distinctly separated from the projection of the 4GBZ structure by the values of PC2 (Fig. 3A).

### Small TM tilt angle changes are associated with IF to OF transition

**Figure 5:**
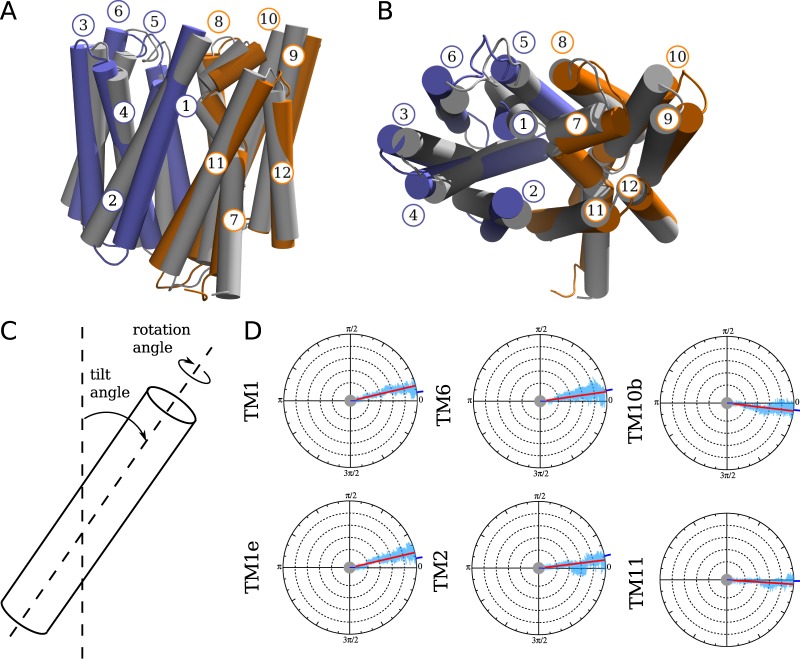
Superposition of the TM part of the inward and outward facing structures of the GluT1 model shown in gray and in color respectively in the side (**A**) and top (**B**) views. In (**C**), the geometrical parameters used for the analysis of the TM helix conformations are shown. In (**D**), the values of the rotation angle calculated for the TM helices are plotted as a function of time for one of the MD simulations of GluT1 WT (Fig. S1A, blue line). The angle is measured with respect to the initial inward facing structure. The blue ticks in the origin and on the side of the circles indicate the angle value for the first and last trajectory frames respectively, the red line corresponds to the mean angle value along the trajectory. The plots are shown only for the helices undergoing the most pronounced rotational movements, the complete set of plots can be found in Fig. S5.

GluT1 transition from the inward to the outward facing state is associated with the rearrangement of its TM part (Fig. 5A,B). In order to provide the detailed view of the observed transitions, we have split the MD trajectories into the fragments for which GluT1 retains either outward facing or inward facing conformation and calculated the average geometrical parameters of each TM helix (Table 1, Figs 5D and S5). We considered two main parameters defining the helix global conformation: the helix tilt angle measured as the angle between the helix inertia axis and the normal vector to the membrane plane, and the angle of the rigid body rotation of the helix around its inertia axis (Fig. 5C).

**Table 1:** Tilt angle values of the GluT1 TM helices during MD simulations in holo and apo states calculated for the inward facing (IF) and outward facing (OF) conformations (degrees).

	Apo (IF)	Holo (IF)	Apo (OF)	Holo (OF)
TM1	34.7 ± 1.5	37.7 ± 3.4	29.1 ± 1.1	34.9 ± 3.4
TM1e	57.2 ± 1.8	60.0 ± 3.6	55.8 ± 1.8	57.9 ± 3.6
TM2	23.1 ± 0.7	26.0 ± 3.2	18.9 ± 0.7	19.3 ± 3.0
TM3	3.3 ± 1.0	8.0 ± 2.8	1.9 ± 0.9	5.1 ± 2.4
TM4	24.7 ± 0.9	27.4 ± 2.9	23.8 ± 0.7	25.0 ± 2.9
TM5	7.0 ± 1.3	7.4 ± 3.0	10.5 ± 1.1	8.9 ± 3.1
TM6	17.3 ± 0.9	21.4 ± 3.3	12.8 ± 1.0	19.4 ± 3.1
TM7a	32.5 ± 2.2	28.6 ± 3.0	34.3 ± 1.4	30.1 ± 3.6
TM7b	30.1 ± 1.5	29.0 ± 3.5	28.6 ± 2.0	26.1 ± 3.9
TM8	13.8 ± 1.1	13.3 ± 2.5	12.8 ± 2.3	13.8 ± 3.0
TM9	9.1 ± 1.1	12.5 ± 3.2	10.4 ± 1.2	13.1 ± 3.3
TM10a	21.2 ± 1.2	21.1 ± 3.2	20.9 ± 1.5	20.9 ± 3.4
TM10b	33.1 ± 3.0	28.7 ± 3.6	32.0 ± 2.1	39.3 ± 3.7
TM11	19.4 ± 0.8	22.4 ± 3.2	21.6 ± 0.9	31.7 ± 3.3
TM12	6.4 ± 1.3	4.8 ± 2.4	2.8 ± 1.0	5.8 ± 2.8

The most pronounced difference in tilt angles between the outward facing and inward facing conformations concerns helices TM1, TM2, TM6 and TM11 (Table 1, Fig. 5A,B). The TM1, TM2 and TM11 helices contour the GluT1 cavity and thus directly contribute to its opening towards the extra- and intracellular medium, while TM6 mobility comes from its interaction with TM1. Most of the residues forming the transmembrane part of TM1, TM2 and TM6 bring small side chains, which allows their rotation detected in our simulations (Fig. 5C,D). TM11 rotation is in turn limited by the presence of the bulky residue Trp412 located close to the cavity center. As the result, it retains the initial orientation and the rotation angle fluctuations do not exceed ~10° similar to the other TM helices (Fig. S5).

The pronounced rotational movements are also present at the extra- and intracellular sides of the TM helices controlling the entrances to the protein cavity: TM1e (Fig. 1) and TM10b (Fig. 5D). The rotation of TM1e impacts its interaction with TM7b locking the extracellular gate (see the next section). The rotation of TM10b helix fragment is in turn necessary for opening of the intracellular gate of the protein. At the same time, in the absence of the ligand we did not observe any prominent rotation of the extracellular part of TM7 helix previously identified on the basis of the structural analysis^11^.

### Intra- and extracellular domains are major actors in GluT1 mobility and are strongly coupled

**Figure 6:**
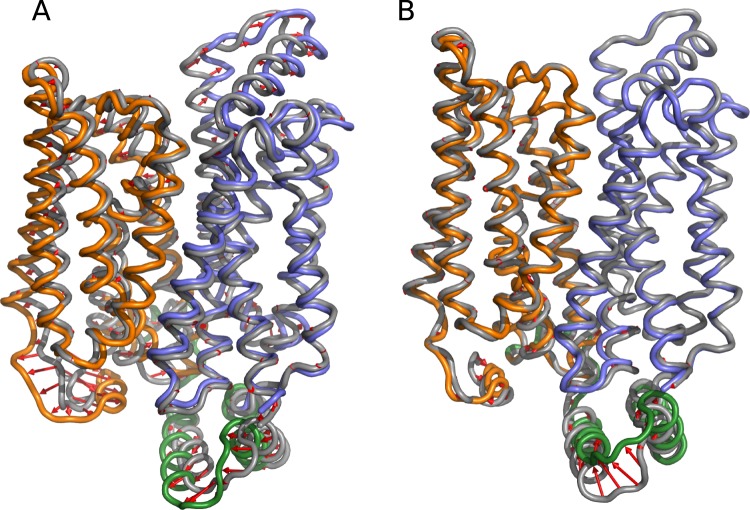
The movements associated with the first (**A**) and second (**B**) PCs calculated for the GluT1 MD simulations. N-terminal, C-terminal and intracellular domains are colored in blue, orange and green respectively. Red arrows indicate the most important conformational changes.

While the TM helices rearrangement is required for the conformational transition of the protein, the intra- and extracellular domains play a crucial role in this process. In order to describe the global conformational transitions observed in our model, we have performed PCA for the GluT1 conformers adopted during MD simulations. The most pronounces conformational changes described by the first PC (48.9% of the overall variance) correspond to the transitions at the level of the extracellular part of TM1, TM2 and TM11 and rearrangement of the IC part of the protein (Fig. 6A). The second PC (16.8% of the overall variance) mostly concerns the displacement of IC2 and IC3 (Fig. 6B).

**Figure 7:**
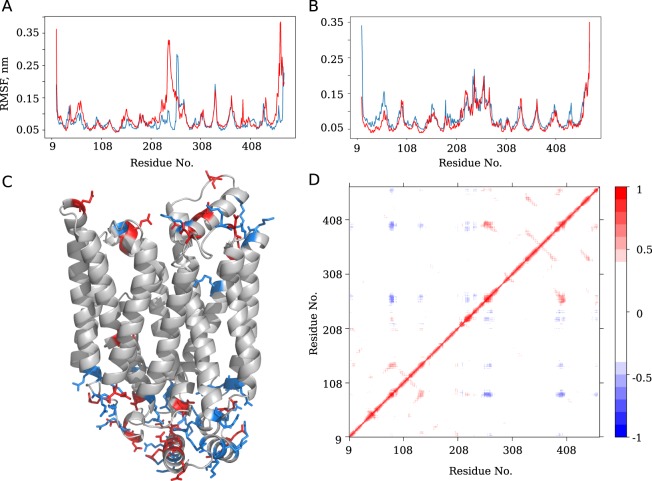
Root mean square fluctuations (RMSF) calculated for the C_*α*_ of the protein in the outward facing (**A**) and inward facing (**B**) conformations in the absence (blue lines) and presence (red lines) of glucose. In (**C**), the outward facing structure of GluT1 with positively and negatively charged residues shown in blue and red respectively. In (**D**), the matrix of cross-corelations between all the residues of the protein calculated for the MD simulations. Red and blue colors correspond to the correlated and anticorrelated movements respectively.

During all the MD simulations intra- and extracellular domains demonstrate the highest conformational mobility, as can be seen from the RMSF profiles (Fig. 7A,B). The maximal RMSF values correspond to residues 200–275 covering the IC domain. In the TM part of the molecule, the most mobile regions are located in TM1e (residues 35–70), and at the ends of helices TM2 (residues 80–90) and TM4 (residues 140–150).

The observed large amplitude movements appear to be coupled between each other. The cross-correlation map developed for the free GluT1 dynamics (Fig. 7D) reveals correlated movements between the intracellular end of TM7 and IC4 (residues 250–280) and the intracellular ends of TM10 and TM11 (residues 380–410). The movements of IC4, TM7, TM10 and TM11 located in the C-domain are correlated with the movements of the extracellular part of TM2 and TM4 of the N-domain.

Anticorrelated movements in turn involve mostly intracellular portions of the N- and C-domains. The most important movements concern the intracellular parts of TM4 (residues 140–150), which are anticorrelated with the movements of TM7 (residues 265–280) and TM10b and TM11 (residues 385–405).

Interestingly, the movements of IC5 (Fig. 1) are strongly correlated with those of the intracellular portion of the C-domain, in particular with the ends of TM7 (residues 260–270), TM8 (residues 325–335) and TM10 (residues 385–395). IC5 also appears to be essentially involved in the anticorrelated movements with the intracellular end of TM2 of the N-domain (residues 80–85) and the intracellular helices IC1 to IC3 (residues 205–255).

High mobility of IC5 comes from the fact that it is significantly involved in the protein cavity closure from the intracellular side. Indeed, in our simulations, the GluT1 transition from the closed inward facing to the outward facing state is accompanied by the IC5 movement towards the cavity entrance and formation of the strong interactions with the other IC helix from the N-domain (IC3). This finding is in accordance with the experimentally predicted role of C-terminus in the GluT1 state transition^23–25^.

#### A tightly connected salt bridge network controls the transition

**Figure 8:**
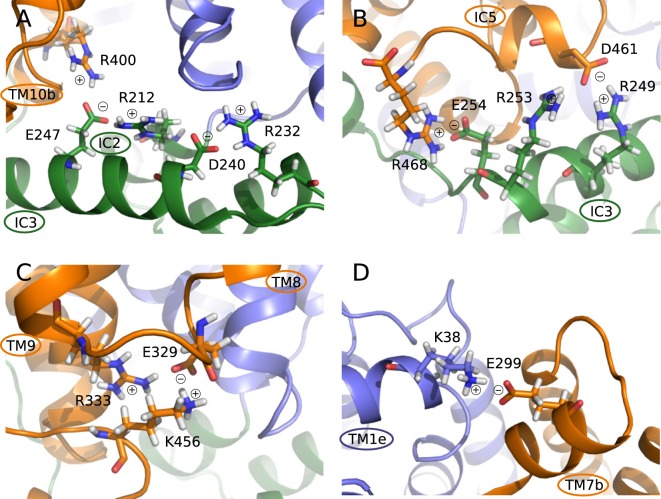
Salt bridges formed between the GluT1 side chains during MD simulations at the intracellular (**A**–**C**) and extracellular (**D**) parts of the protein. C- and N-domains are colored in blue and orange respectively, the intracellular helices are colored in green.

Most of the charged residues are located at the ends of the TM helices and at the IC part of the protein (Fig. 7C). This leads to the formation of the strong network of salt bridges at the intracellular side of the protein observed in MD simulations (Fig. 8A–C, Table S2). First of all, IC helices are strongly connected to each other during all the simulations by Asp240-Arg232 salt bridge between IC2 and IC3 and by Glu247-Arg212 between IC3 and IC1 (Fig. 8A). IC1 is in turn connected to the intracellular portion of TM3 by Glu209-Arg93, while TM3 also forms a strong salt bridge with TM4 (Glu146-Arg92). Finally, intracellular ends of TM10 and TM9 strongly interact by Glu393-Arg333. The C-terminus is connected to the intracellular portion of TM8 by Glu329-Lys456 salt bridge.

During our simulations, Glu329 formed multiple salt bridges: with Arg153, Arg330, Arg333 and Lys456 (Fig. 8C). The initial hypothesis for the mutation of Glu329 residue to glutamine for GluT1 crystallization in the inward open conformation relied on its supposed implication to the formation of the hydrogen bonds with amide groups of Gly154 and Ala15 of the N-domain during transition to the closed state^12^. During our simulations we have not observed such interactions and Glu329 is mostly involved in the salt bridge formation with TM helices and C-terminus. Thus, we suggest that the key role of Glu329 would be stabilization of the C-terminus necessary for the closure of the protein cavity from the intracellular side^23–25^.

In the outward facing conformation the IC3 helix is also involved in the interactions with the TM10 intracellular end (Arg400-Glu247 in Fig. 8A) and forms a set of salt bridges with C-terminus (Asp461-Arg249, Asp461-Arg253, Arg468-Glu254 in Fig. 8B). Such developed network of electrostatic interactions with participation of IC3 confirms its role of the “door closer” in maintaining the outward facing conformation of GluT1^11^.

We have also observed several very stable salt bridges formed at the extracellular side of the protein. Glu120-Arg51 keeps TM1e helix (Fig. 1) attached to the extracellular end of TM4, TM11 is connected to TM7 by Glu426-Lys300 interaction and Glu299-Lys38 connects TM1e and TM7 (Fig. 8D).

### Glucose transit along GluT1 through three main stages

**Figure 9:**
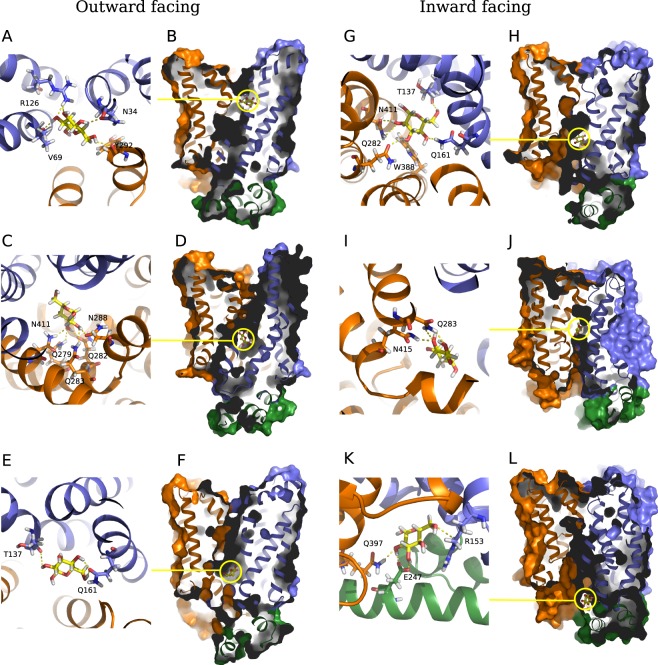
Snapshots of the MD simulations of glucose interaction with GluT1 in the outward facing (**A**–**F**) and inward facing conformations (**G**–**L**). The residues of N- and C-terminal domains are colored in blue and orange respectively, intracellular helices are colored in green, the glucose molecule is colored in yellow.

The obtained in MD simulation outward facing structure of GluT1 WT was further used to model the glucose transport by the protein. We have performed docking of the glucose molecule to the extracellular pocket of GluT1 introducing the flexibility of the side chains at the extracellular side of the protein cavity (see Methods). The resulting energetically favourable positions of glucose were used as starting points for the MD simulations. In most of the cases glucose was leaving the transporter instead of entering the protein cavity. The only starting conformation for which we succeeded to reproduce the glucose uptake by the transporter corresponds to the structure with glucose entering the extracellular pocket between TM1,TM2 and TM4 of the N-domain and TM7,TM11 of the C-domain about 13 Å away from the central cavity reported in literature (Fig. 9B). In this position, glucose forms strong hydrogen bonds with four neighbouring residues: Val69, Arg126, Asn34, Tyr292 (Fig. 9A). The similar binding site was previously reported to be a ligand recognition pocket according to docking studies^20^. Moreover, the mutation in Arg126^26–28^ and Asn34^29^ are related to the decrease of the glucose flux associated with the Glut1 deficiency syndrome. Both residues are conserved among MFS family members (GluT3, XylE), and mutation in Arg126 is the most frequent mutation observed in patients with this pathology.

After ~2.5 ns equilibration in the binding site, we have observed glucose rotation by 60° leading to the formation of hydrogen bonds with Thr30 (TM1), Ser73 (TM2), Tyr292 (TM7) and Asn415 (TM11), which stabilize glucose in this site for almost 4 ns. Accompanied by the rotation of Tyr292 glucose shifts for ~5 Å to the position stabilized by hydrogen bonds with Asn288, Asn411 and Gln283 and further towards to the extracellular part of the protein cavity to the quasi-equilibrium point stabilized by Asn411, Asn288, Gln279, Gln282, Gln283 (Fig. 9C,D) and eventual interaction with Asn415 and Trp412. Weakening of the hydrogen bond between glucose and Tyr292 due to the glucose rotational movements allows the Tyr292 to adopt the conformation with aromatic ring parallel to the membrane plane. As the result, it locks the extracellular entrance to the protein cavity, which leads to the GluT1 transition to the closed state.

#### “Rock’n’roll” motions of glucose allows its progression along the cavity

The closed conformation appears to be a favorable state of the protein in the presence of solute. We have observed fluctuation of the glucose molecule inside the GluT1 cavity for more than 1 *μ*s. Glucose movement is governed by the side chain translocation but also by the formation of hydrogen bonds with oxygen atoms of the main chain. We have estimated the stability of the hydrogen bonds formed between glucose and the GluT1 residues by calculation of the maximal time period of their existence during MD simulations (Table 2). The most populated states concern the described binding site (Fig. 9C,D) and the glucose position at the bottom of the cavity characterized by formation of strong hydrogen bonds with side chain atoms of Gln161 (Fig. 9E) and interaction with Trp388, Gln282, His160 as well as by formation of the hydrogen bonds with the oxygen atoms of the main chain for His160, Thr137 and Ile404.

**Table 2:** Maximal time period of the hydrogen bond interaction between glucose molecule and GluT1 residues in the outward facing and inward facing conformations calculated for the atoms of the main chain and side chains during MD simulations (ns).

Main chain			Side chain		
Res.	Outward-facing	Inward-facing	Res.	Outward-facing	Inward-facing
Thr137	5.6	0.9	Ser23	7.4	—
Pro141	—	1.5	Thr30	4.3	1.3
Gly145	—	1.7	Asn34	1.5	—
Arg153	—	0.8	Ser73	0.7	—
Gly157	1.2	—	Arg126	0.6	—
His160	4.6	—	Thr137	2.3	—
Gly384	2.2	3.2	His160	0.5	2.6
Ala392	—	1.1	Gln161	17.0	10.5
Ile404	1.9	—	Arg212	—	0.5
Ala407	0.5	—	Glu243	—	0.9
			Glu247	—	3.7
			Gln279	6.3	—
			Gln282	6.5	19.1
			Gln283	5.5	7.4
			Asn288	2.7	2.9
			Tyr292	1.1	1.1
			Glu380	—	13.6
			Trp388	0.6	4.0
			Glu393	—	1.7
			Gln397	—	0.8
			Arg400	—	2.1
			Asn411	6.6	10.8
			Asn415	6.2	1.1

Only the residues interacting with glucose for more than 500 ps are listed.

In the inward facing closed GluT1 conformation glucose spends several nanoseconds fluctuating between the different intermediate binding pockets (Fig. 9I,J), then finally passes the Trp388 barrier and enters a region located closer to the intracellular side of GluT1 surrounded by hydrophobic residues Met142, Gly157, Gly145 and Phe389, and interacting with the main chain oxygen atoms of Ile404, Pro141 and Thr137. These interactions slow down glucose progression towards the intracellular part of the protein which is in accordance with the local free energy minima previously predicted for this region on the basis of the steered MD simulations^19^.

Glucose hydrogen bond formation with Arg400 and Glu393 leads to the reorientation of the hydrophobic side chains. As the result, glucoses passes to the intracellular binding pocket formed by Glu247, Arg153 and Glu397 (Fig. 9K,L), and finally leaves the transporter and enters the intracellular medium.

#### Glucose impact on the GluT1 conformational dynamics

Despite the small size and high mobility of the glucose molecule inside the protein, its presence affects the arrangement of the TM part of the transporter. In terms of the protein core projection on the subspace given by the PC determined for the MFS X-ray structures (Fig. 3B), the inward and outward facing conformations correspond to the similar highly populated zones of the conformational space as for the GluT1 molecule in the absence of ligand (Fig. 3A). At the same time, glucose significantly affects the local mobility of the TM helices participating in its transfer. First of all, glucose uptake at the extracellular part of the protein increases the amplitude of the rigid body rotation for TM7b as well as for the extracellular part of TM1 (Fig. S5) forming the extracellular gate and regulating the hydrophobic barrier of the Tyr292 and Tyr293 residues. Then, during the glucose transfer to the protein cavity we observe a slight increase of the amplitude of the tilt angle fluctuations for all the TM helices. The most affected helices are TM2 and the transmembrane part of TM11 (Table 1), which demonstrate the relative tilts during the conformational transition equal to ~4.2° and 2.2° in the presence of glucose as compared to ~6.7° and ~9.3° respectively.

At the level of the intra- and extracellular helices, the presence of glucose molecule affects the properties of the salt bridge network. Directly interacting with the charged residues, glucose causes the disruption of the Glu299-Lys38 salt bridge while entering the protein cavity from the extracellular side and also disrupts the Lys256-Glu247 salt bridge during the exit at the intracellular side.

## Discussion

In the current study we have addressed the mechanism of the conformational transitions of the human glucose transporter GluT1 in the absence and in the presence of solute. The recently resolved GluT1 X-ray structures were obtained only for the protein conformation open to the cytoplasm using different ligands for its stabilization^12,13^. According to the accepted general transport mechanism of the MFS proteins^4^, GluT1 should adopt the similar conformation during the glucose release to the intracellular medium. At the same time, the glucose uptake from the extracellular medium requires protein transition to the outward-facing conformation, whose crystal structure was never obtained for GluT1. In the current study we for the first time reproduce this transition in classical molecular dynamics simulations.

In the previous model studies of the GluT1 transport mechanism the outward facing state of the protein was obtained by homology modelling using as a template the structure of the bacterial glucose transporter XylE^19,20,30^. Nevertheless, the arrangement of the transmembrane parts of GluTs and their bacterial homologs is not exactly the same. According to the results of the principal component analysis performed for the common transmembrane part of the available MFS protein X-ray structures, GluTs adopt conformations distinctly separated from the XylE structures by the values of the second PC. This difference is further confirmed by the projection of the GluT1 conformers obtained in MD simulations on the subspace formed by the first two PC. In our simulations starting from the inward open crystal structure, GluT1 undergoes transition to the outward facing state due to the simple atomic fluctuations. The resulting conformation is very close to the GluT3 X-ray outward facing structure (PDB ID: 4ZW9) but remains separated from the XylE conformers. Moreover, all the intermediate closed states adopted by the protein during MD simulations never visit the area of conformational space corresponding to the X-ray structures of the GluT bacterial homologs. Taking all this into account, we suggest that the XylE outward facing conformation must differ from the GluT1 outward facing native structure. This result is also supported by the thermodynamic difference between GluT1 and XylE conformational transitions predicted by the comparative analysis of the accelerated MD simulations^31^.

All the obtained in MD simulations conformers demonstrate lower degree of opening to the extracellular side as compared to the outward open X-ray structure of XylE both in terms of the size of the extracellular part of the protein cavity and TM helices arrangement. The inward open X-ray structure also appears to be an unfavourable conformation and undergoes fast transition to the more closed state during MD simulations. Nevertheless, using the conformers obtained in MD simulations, we succeed to reproduce the glucose uptake by the protein, its transport in the protein cavity and finally its release to the intracellular medium. Thus, we can expect that in real membrane systems the solute transport by GluT1 requires more modest transitions of the protein global conformation than observed in the available X-ray crystal structures.

The analysis of the MD trajectories reveals significant inclination of the TM helices 1,2,6 and 11 as well as pronounced rotation of TM1, TM2 and TM6 during the protein state transition. During the glucose transfer, the reorientation of the TM helices is accompanied by the local translocation of the side chains exposed to the protein cavity. Most of the residues involved in the interaction with glucose such as Q161 on TM5, Q282/Q283/N288/Y292 on TM7, G384/W388 of TM10 are conserved among the SP MFS proteins^32^. At the same time, residue Asn411 (TM11) highly involved in glucose transport, is substituted by His and Gln in GluT5 and XylE respectively, which can affect their binding affinity.

GluT1 transition between the outward and inward facing states is significantly affected by the movements at the intra- end extracellular parts of the protein. IC region (residues 211–271) demonstrates high conformational mobility during all the simulations. These movements are anti-correlated to the movements of the extracellular part of TM7, that governs the formation of the endofacial pocket. The important contribution of the intra- and extracellular parts of the protein to the GluT1 structural mobility can be explained by the role of electrostatic interactions in the energy transfer during internal structural rearrangements^33^. Indeed, both intra- and extracellular helices bring high concentration of the charged residues and form a network of salt bridges stabilizing the protein conformation. The IC helices form numerous salt bridges both between each other and with the intracellular ends of the TM helices. The mutations of some of the involved charged residues (R333W^34^, E299K^29^, E247D^32^, R249A/R400H/R400C/R153C^35^, R153G/R223T/R249A/R223W^36^) are associated with the GluT1 deficiency syndrome. The disruption of the salt bridge at the extracellular part of the protein between TM1e (Fig. 1) and TM7 (Glu299-Lys38) due to the K38A mutation leads to the dramatical decrease of the glucose uptake^30^.

Finally, the glucose transfer accompanied by the protein transition from the outward facing to the inward facing state is a highly cooperative process and requires simultaneous rearrangement of the local and global protein structure induced by a solute. In the work of Park *et al*.^19^, the protein conformational transition during the glucose transfer was induced by addition of the external force on the protein C_*α*_ atoms in targeted MD simulations. Fu *et al*. followed a different protocol and performed a set of MD simulations starting from the distinct conformational states of the protein with glucose binding sites identified by docking procedure for each of these states^20^. During the relatively short 60 ns simulation time no transition between the identified discrete states was observed^20^. In contrast to the previous studies, the long MD simulations of GluT1 in presence of glucose performed in the current work allows us to reproduce the continuous solute translocation in the protein and simultaneously estimate its impact on the conformational dynamics of the transporter. We note that the residence time of the glucose in the GluT1 central cavity is rather long in regards to the total simulation time. Once entering the protein cavity, the glucose interacts with many residues by forming multiple hydrogen bonds, which contribute to a favourable enthalpic interaction. Meanwhile, the glucose undergoes numerous rotations and axial movements, which might result in a favourable entropic contribution. Overall, the free energy of glucose binding in the protein cavity might be lower compared to other ligand positions and slow down the process of the glucose transfer to the the intracellular medium. Nevertheless, the results of our simulations confirm that the GluT1 state transition does not require any additional energy input, which is in accordance with the facilitative uniporter transport mechanism.

## Methods

### GLUT1 structure

The original GluT1 structure was obtained from the Protein Data Bank (PDB) under the 4PYP code^12^. We removed the ligand present in the X-ray structure (*β*-nonylglucoside) and oriented the protein molecule in the lipid bilayer using the PPM server^37^. Hydrogen atoms were added using pdb2gmx GROMACS utility^38^ and the residues were protonated to mimic their state of protonation at physiological pH of 7.4 on the basis of the pK data PROPKA^39^.

The 4PYP structure contains two mutations (N45T and E329Q). The first mutation, N45T removes a glycosylation site and allows to express a more homogeneous protein. The second mutation, E329Q, is intended to lock the protein in the open to the cytoplasm conformation^12^. In order to obtain the WT model we have inverted both mutations using the PyMOL mutagenesis tool. Finally, the 4PYP crystal structure lacks a fragment of the C-terminal domain (residues 452–462) and its structure was completed by comparison with the corresponding fragment of the 4GBZ structure.

### Molecular dynamics simulations protocol

GluT1 molecule was inserted into palmitoyl-oleoyl phosphatidyl choline (POPC) bilayer using the CHARMM-GUI tool^40^. The resulting system was explicitly solvated using the TIP3P water model^41^ and neutralized by the addition of counterions of K^+^ and Cl^−^ ions at concentration of 0.15 M.

All the molecular dynamics (MD) simulations were performed with GROMACS 4.6.5^38^ using the all-atom force field CHARMM36^42^. We used periodic boundary conditions. The long-range electrostatic interactions were treated using the Particle Mesh Ewald (PME) method. A Lennard-Jones potential with a cut-off distance of 1 nm was used to describe the non-bonded interactions. The lengths of the bonds between hydrogen atoms and heavy atoms was fixed using the LINCS algorithm^43^, which allowed us using an integration step of 2 fs. Water molecule structure was maintained using rigid SETTLE algorithm^44^.

Optimization of the model system geometry was performed using the steepest descent algorithm of energy minimization (5000 steps) keeping the positions of the heavy atoms of the protein fixed by harmonic potential (force constant of 1000 kJ/mol/nm). The resulting system was heated to the temperature of 300 K for 1 ns in the NVT ensemble using Berendsen algorithm^45^. Then, for each system we performed 25 ns equilibration simulation in the NPT ensemble restraining heavy atom positions for the protein molecule. The simulations were performed at 300 K with separate temperature coupling for the protein, lipids and solvent molecules using the Nose-Hoover algorithm^46,47^ with a coupling constant *τ*_*T*_ = 0.5 ps. The normal pressure was maintained by the semi-isotropic coupling using the Parrinello-Rahman barostat^48^ with a coupling constant *τ*_*P*_ = 5.0 ps. The resulting equilibrated structures were then used as an initial condition for the production runs of 1–1.5 *μ*s with all the constraints turned off.

### Glucose transport model

#### Molecular docking

We have considered GluT1 transport of *β*-D-glucose molecule. To position the glucose in the binding site of the GluT1 outward facing conformer, molecular docking was performed with the Autodock 4.2.6 software^49^. The input files for docking calculations were obtained using mgltools 1.5.6 software. The GluT1 structure contains only polar hydrogen atoms. We have introduced flexibility of three side chains for the residues located at the entrance of the extracellular protein cavity (Asn34, Tyr292 and Tyr293) in order to prevent glucose blocking and allowed rotation of all the single bonds of glucose to change the ligand conformation during the simulation. Gasteiger partial charges were assigned for all the atoms of the system by default. Conformational search was performed on a grid of 60 × 60 × 60 points separated by 0.375 Å using the Lamarckian genetic algorithm (LGA) with a population size of 300 randomly placed individuals and maximum number of $2.5\cdot {10}^{7}$ energy evaluations.

#### MD simulation of glucose transport

The results of glucose docking to the GluT1 extracellular pocket were used as initial states for the MD simulation of the glucose uptake by the protein. After the solute passed the extracellular gate and entered the protein cavity, its further transfer to intracellular part of the protein is prevented by the hydrophobic barrier formed by Trp388. Its opening implies an important displacement of the intracellular part of TM10^19,20^. In our conventional MD simulations the glucose fluctuation in the central cavity does not induce the required transition. Thus, in order to reproduce glucose displacement to the endofacial pocket, we have considered a model of the inward facing GluT1 reconstructed on the basis of 4PYP X-ray structure (see Methods) and replaced the sugar moiety of the ligand by glucose molecule. Since the X-ray inward open conformation of GluT1 is not stable, we observed fast protein transition to the occluded state with glucose occupying the binding site similar to the outward facing occluded state (Fig. 9G,H as compared to C,D). Moreover, in the resulting occluded structure is very close to the occluded state obtained starting from the outward facing conformation in terms of the projection on the subspace of the MFS conformers (Fig. 3B) with the RMSD calculated for the C_*α*_ atoms of the core equal ~1.2 Å. Since transition between such close conformers was observed during MD simulations for the ligand-free GluT1 model, we considered the obtained conformation to model glucose exit to the intracellular medium.

### Analysis of the MD trajectories

We used the Bio3d tool^50^ to perform a principal component analysis (PCA) of the set of the MFS protein structures previously superimposed by C_*α*_ atoms of the common core. We have considered 14 X-ray structures of the MFS proteins belonging to the SP family. Several monomers from the same crystal were considered to be identical if the overall RMSD value lie below 1 Å. The common core of the selected structures was identified by performing structural alignment using Matt software^51^. The subspace formed by the two first PCs was then used for analysis of the GluT1 conformations during MD simulations.

Detection of salt bridges in the GluT1 structure was performed using the PyInteraph tool^52^ with the threshold distance between the concerned residue parts equal 4 Å.

Analysis of the cross-correlations matrix of atomic fluctuations was performed using the Bio3d tool^50^ by taking into account the C_*α*_ conformers from the simulations. Structures have been extracted from the trajectories every 100 ps and a covariance matrix was constructed for each residues *i* and *j*.

Analysis of the hydrogen bonds formed between glucose and protein molecule was performed using the GROMACS utility hbnum. The RMSF values and values of the tilt angles of TM helices were calculated with help of the GROMACS utilities. Analysis of the rotational movements of TM helices was performed with help of the TRAJELIX tool^53^ of the Simulaid package^54^.

## Supplementary information

New insights into GluT1 mechanics during glucose transfer

## Data Availability

The datasets generated during the current study are available from the corresponding author on reasonable request.
