# Supplementary material for: New insights into GluT1 mechanics during glucose transfer
Source: Sci Rep. 2019 Jan 30;9:998. doi: 10.1038/s41598-018-37367-z (PMC6353926; doi:10.1038/s41598-018-37367-z)
Supplement: Supplementary file 1 — New insights into GluT1 mechanics during glucose transfer [file 41598_2018_37367_MOESM1_ESM.pdf]

New insights into GluT1 mechanics during glucose transfer

Tatiana Galochkina[1,+], Matthieu Ng Fuk Chong[1,+], Lylia Challali[1], Sonia Abbar[1], and Catherine Etchebest[1,*]

[1]Université Sorbonne Paris Cité, Université Paris Diderot, Inserm, INTS, Unité Biologie Intégrée du Globule Rouge UMR_S1134, DSIMB, Laboratoire d'Excellence GR-Ex, Paris, 75739, France
[*]catherine.etchebest@inserm.fr
[+]these authors contributed equally to this work

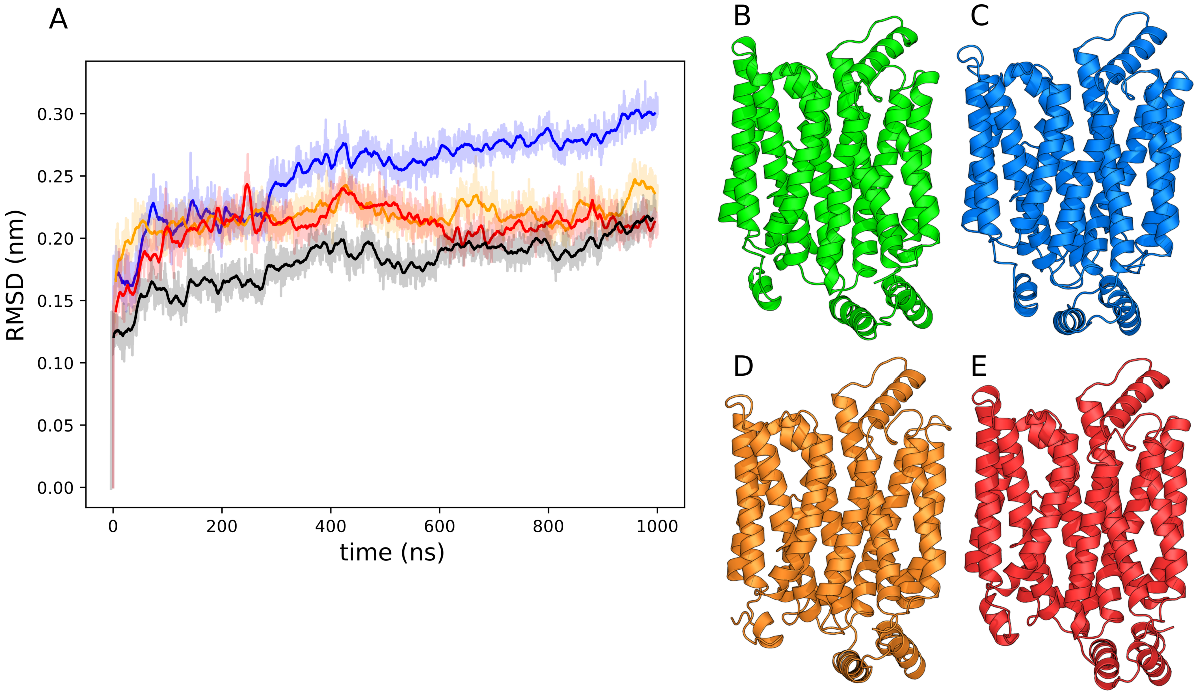

Figure S1. The RMSD calculated for C_α atoms in three replicas of the MD simulation of GluT1 WT in the lipid bilayer (A), the reference X-ray structure (B) and the final protein conformations for each replicate (B,C,D) coloured similar to the corresponding RMSD plots. The blue line corresponds to the simulation when GluT1 undergoes transition from the inward facing to the outward facing state. For this trajectory we also provide the RMSD values calculated for the transmembrane part of the protein (black line).

Table S1. Available X-ray structures of the SP MFS proteins.

Protein	Function	Organism	PDB ID	Resolution (Å)	Conformation	Ligand	Ref.
GLUT1	D-glucose transporter	*Homo sapiens*	4PYP	3.17	Inward open	β-Nonyl-glucoside	1
			5EQG	2.9	Inward open	Glut inhibitor ($C_{25}H_{25}FN_2O_3$)	2
			5EQH	2.99	Inward open	Glut inhibitor ($C_{26}H_{27}BrN_2O_3$)	2
			5EQI	3.0	Inward open	Cytochalasin B	2
GLUT3	D-glucose transporter	*Homo sapiens*	5C65	2.65	Outward open	—	—
			4ZW9	1.50	Partly occluded, outward-facing	β-D-Glucose, α-D-Glucose, 1-Oleoyl-R-glycerol	3
			4ZWB	2.40	Partly occluded, outward-facing	Maltose	3
			4ZWC	2.60	Outward open	Maltose	3
GLUT5	D-fructose transporter	*Bos taurus*	4YB9	3.20	Inward open	—	4
		Rattus norvegicus	4YBQ	3.27	Outward open	—	4
XylE	D-xylose-proton symporter	*Escherichia coli*	4GBY	2.81	Partly occluded, outward-facing	β-D-Xylose	5
			4GBZ	2.68	Partly occluded, outward-facing	β-D-Glucose	5
			4GC0	2.60	Partly occluded, outward-facing	6-bromo-6-deoxy-β-D-Glucose	5
			4JA3	3.80	Partly occluded, inward-facing	—	6
			4JA4	4.20	Inward open	—	6
			4QIQ	3.51	Inward open	—	7
GlcP	D-glucose-proton symporter	*Staphylococcus epidermidis*	4LDS	3.20	Inward open	—	8

References

1. Deng, D. *et al.* Crystal structure of the human glucose transporter GLUT1. *Nature* **510**, 121–125 (2014). DOI 10.1038/nature13306.
2. Kapoor, K. *et al.* Mechanism of inhibition of human glucose transporter GLUT1 is conserved between cytochalasin B and phenylalanine amides. *Proc. Natl. Acad. Sci.* **113**, 4711–4716 (2016).
3. Deng, D. *et al.* Molecular basis of ligand recognition and transport by glucose transporters. *Nature* **526**, 391–396 (2015).
4. Nomura, N. *et al.* Structure and mechanism of the mammalian fructose transporter GLUT5. *Nature* **526**, 397–401 (2015).
5. Sun, L. *et al.* Crystal structure of a bacterial homologue of glucose transporters GLUT1-4. *Nature* **490**, 361–366 (2012). DOI 10.1038/nature11524.
6. Quistgaard, E. M., Löw, C., Moberg, P., Trésaugues, L. & Nordlund, P. Structural basis for substrate transport in the GLUT-homology family of monosaccharide transporters. *Nat. Struct. Mol. Biol.* **20**, 766–768 (2013). DOI 10.1038/nsmb.2569.
7. Wisedchaisri, G., Park, M. S., Iadanza, M. G., Zheng, H. & Gonen, T. Proton-coupled sugar transport in the prototypical major facilitator superfamily protein XylE. *Nat. Commun.* **5**, 1–11 (2014). DOI 10.1038/ncomms5521.
8. Iancu, C. V., Zamoon, J., Bum, S., Aleshin, A. & Choe, J.-y. Crystal structure of a glucose / H + symporter and its mechanism of action. *Proc. Natl. Acad. Sci. U. S. A.* **110**, 17862–17867 (2013).

Table S2. Salt bridges observed during MD simulations for inward facing (IF) and outward facing (OF) states. Numbers reflect the percentage of the overall trajectory time for which the existence of the salt bridge was observed.

		IF apo	IF holo	OF apo	OF holo			IF apo	IF holo	OF apo	OF holo
41 Glu	38 Lys	75.4	88.2	95.0	80.2	254 Glu	249 Arg	9.5	0.1	–	–
42 Glu	38 Lys	0.3	4.5	2.3	5.4	254 Glu	253 Arg	11.2	24.2	25.6	12.1
114 Lys	42 Glu	0.1	0.9	1.1	0.1	255 Lys	254 Glu	13.2	6.0	3.0	4.1
120 Glu	51 Arg	100.0	100.0	100.0	100.0	256 Lys	247 Glu	20.0	0.6	–	–
146 Glu	92 Arg	99.8	100.0	100.0	100.0	261 Glu	255 Lys	26.7	13.3	2.9	3.8
177 Asp	38 Lys	0.2	0.3	2.8	9.7	264 Arg	254 Glu	–	–	61.0	–
183 Lys	42 Glu	62.9	70.4	79.9	54.0	264 Arg	261 Glu	79.4	99.9	99.9	7.6
184 Asp	114 Lys	82.5	83.4	46.3	81.3	299 Glu	38 Lys	79.4	76.4	89.3	13.8
184 Asp	183 Lys	31.9	26.1	27.1	41.5	300 Lys	299 Glu	4.3	4.8	9.0	10.2
209 Glu	9 Thr	6.9	–	–	–	329 Glu	153 Arg	–	0.3	1.1	17.7
209 Glu	11 Arg	5.2	–	–	–	330 Arg	329 Glu	–	5.3	0.7	14.2
209 Glu	92 Arg	–	–	35.6	0.3	333 Arg	329 Glu	11.3	29.5	58.8	19.2
209 Glu	93 Arg	92.4	96.4	95.7	44.1	393 Glu	153 Arg	0.0	13.9	74.2	94.6
212 Arg	146 Glu	89.8	84.5	0.0	2.0	393 Glu	333 Arg	100.0	100.0	100.0	100.0
218 Arg	209 Glu	1.1	4.4	0.5	2.1	400 Arg	146 Glu	0.1	0.5	57.3	6.0
220 Glu	218 Arg	55.1	78.8	69.1	84.9	400 Arg	243 Glu	50.2	0.1	1.6	–
223 Arg	209 Glu	76.5	97.2	46.7	75.9	400 Arg	247 Glu	13.3	14.5	100.0	0.2
223 Arg	220 Glu	92.3	91.4	94.1	92.0	400 Arg	393 Glu	13.9	1.2	0.1	–
225 Lys	221 Glu	59.1	51.6	51.8	52.0	426 Glu	300 Lys	99.5	99.1	97.9	97.5
230 Lys	209 Glu	1.7	1.8	0.1	7.4	454 Glu	333 Arg	26.4	36.3	27.5	–
236 Asp	232 Arg	–	–	–	19.4	454 Glu	334 Arg	13.9	40.5	34.7	95.8
236 Asp	229 Lys	1.7	3.7	0.8	78.0	456 Lys	243 Glu	–	–	0.0	–
236 Asp	230 Lys	–	–	–	4.7	456 Lys	329 Glu	41.2	50.9	85.5	–
240 Asp	153 Arg	91.4	22.6	17.0	–	456 Lys	393 Glu	47.3	37.6	53.9	–
240 Asp	212 Arg	0.1	6.3	95.2	–	456 Lys	454 Glu	1.7	5.4	0.5	2.7
240 Asp	229 Lys	–	–	–	1.6	458 Arg	243 Glu	–	-	–	1.0
240 Asp	232 Arg	100.0	99.9	100.0	71.0	458 Arg	393 Glu	–	–	–	1.5
243 Glu	153 Arg	99.5	51.2	100.0	0.5	458 Arg	454 Glu	63.0	52.9	20.6	0.0
243 Glu	212 Arg	19.0	82.4	100.0	1.2	461 Asp	153 Arg	–	–	–	43.2
243 Glu	232 Arg	7.9	4.2	–	46.8	461 Asp	249 Arg	0.1	0.1	98.4	24.2
245 Lys	221 Glu	48.5	83.9	7.1	9.8	461 Asp	253 Arg	–	–	9.3	19.6
246 Glu	245 Lys	15.1	1.4	15.8	20.0	462 Glu	249 Arg	–	–	–	1.6
247 Glu	92 Arg	4.9	–	–	–	462 Glu	456 Lys	–	–	–	12.3
247 Glu	153 Arg	1.4	6.8	0.1	10.9	462 Glu	458 Arg	98.2	91.4	99.8	85.5
247 Glu	212 Arg	99.6	100.0	100.0	99.4	468 Arg	253 Arg	–	–	12.3	0.0
247 Glu	232 Arg	–	–	–	46.6	468 Arg	254 Glu	–	0.0	39.6	88.7
249 Arg	221 Glu	0.1	0.7	–	27.7	468 Arg	264 Arg	–	–	1.0	–
249 Arg	246 Glu	93.3	77.5	100.0	64.8	468 Arg	451 Lys	59.6	55.8	–	–
249 Arg	247 Glu	–	–	–	0.1	468 Arg	461 Asp	8.4	10.6	1.3	3.5
253 Arg	221 Glu	7.7	–	–	0.1	468 Arg	462 Glu	0.1	0.1	9.5	
253 Arg	246 Glu	18.0	24.0	–	11.5	468 Arg	468 Arg	83.9	73.8	80.5	71.0

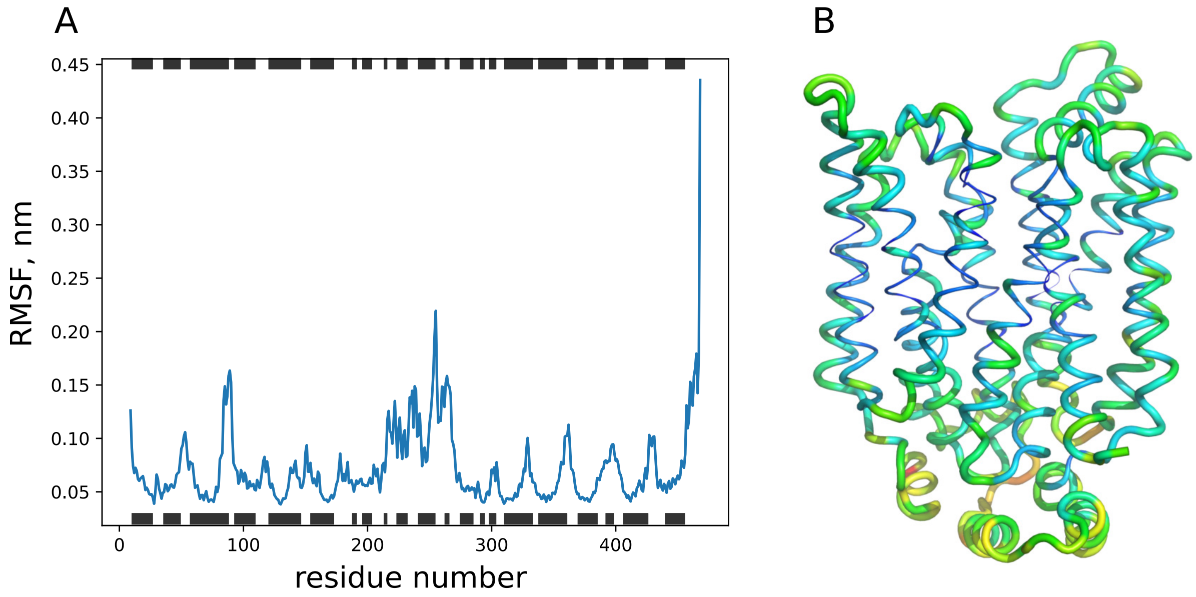

Figure S2. The RMSF of C_α atoms (A) calculated for the GluT1 conformational transition from IF to OF state (time period 200–400 ns in Fig. S1, blue line) and the residue mobility represented on the protein 3D structure (B). The most pronounced conformational changes are observed for the intra- and extra-cellular parts of the protein.

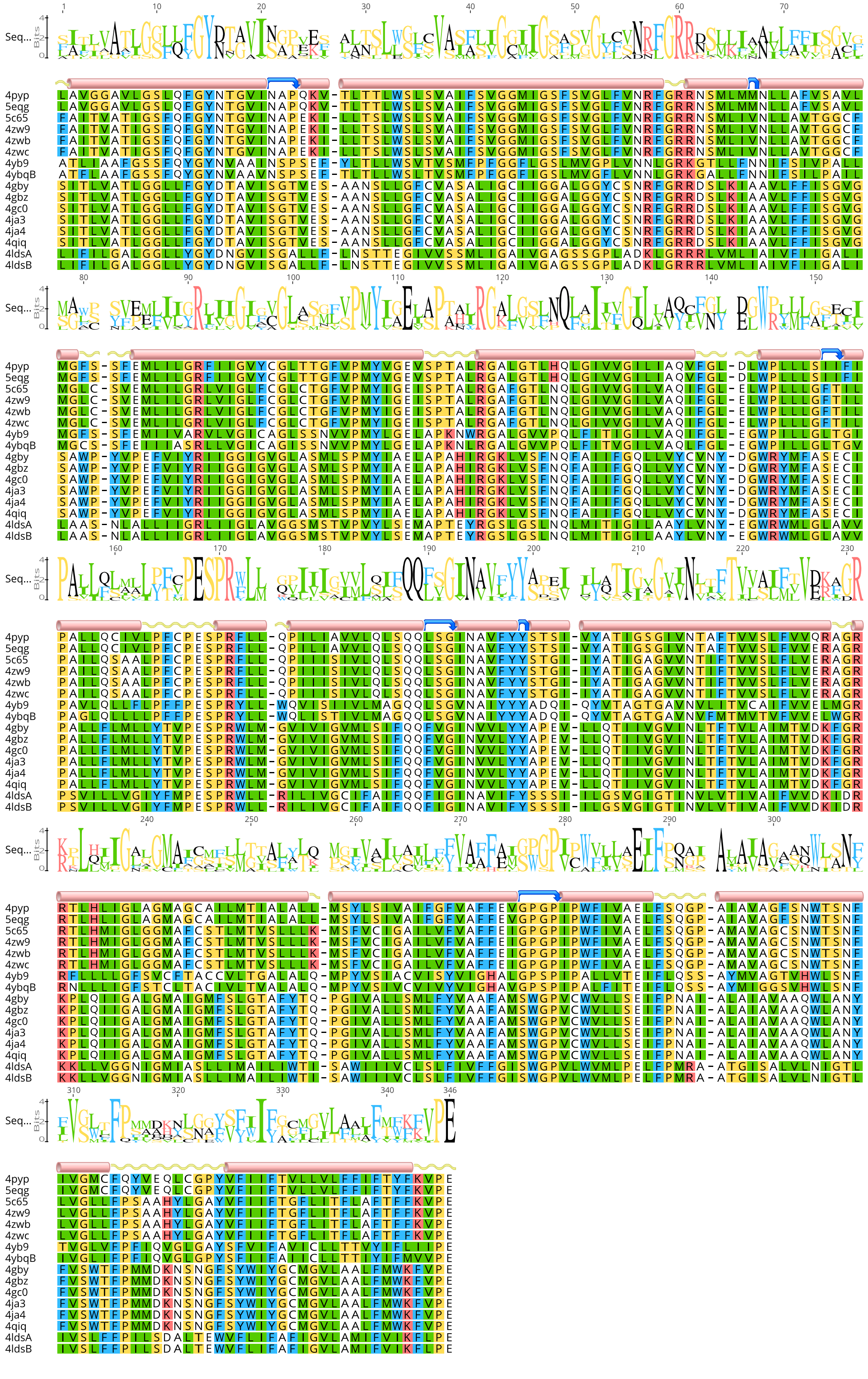

Figure S3. Core part alignment of the crystal structures of the MFS SP proteins (Tab. S1). Residues are colored according to Clustal color scheme, rose cylinders indicate the location of α-helices, connected by the blue arrow if helical fragments make up the same TM helix, yellow strings correspond to the coils between structural fragments.

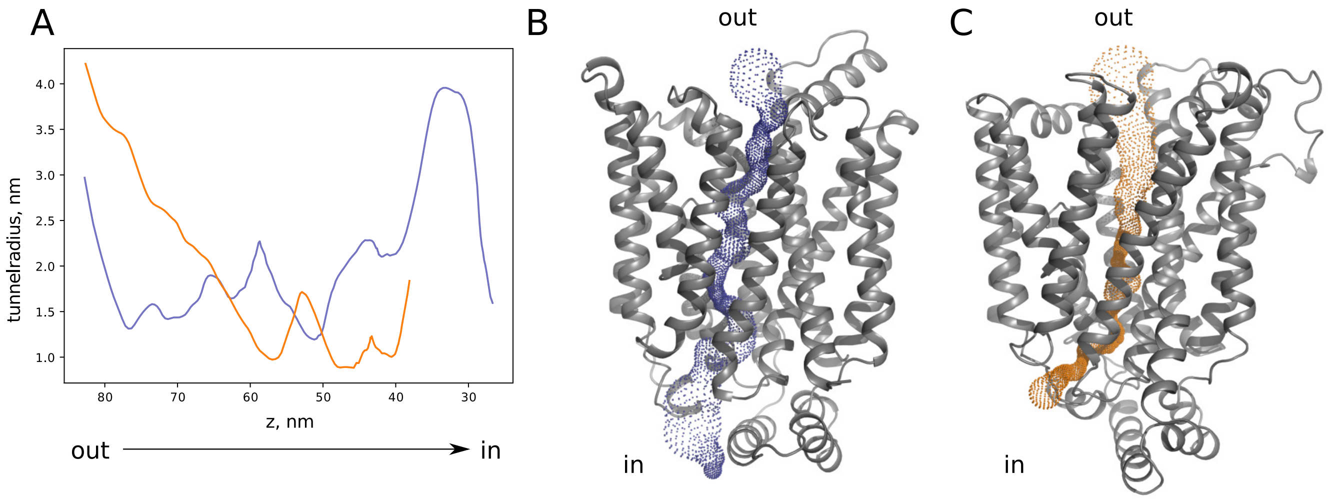

Figure S4. The pathway radius as function of z coordinate (A) calculated for the GluT1 outward facing conformer obtained in MD simulations (blue line) compared to that of the X-ray structure 4GBZ of the XylE outward open conformer (orange line). The corresponding pathways are shown in dots for the GluT1 (B) and XylE (C) structures. Following the extensive studies of the GluT1 protein cavities done by J. Iglesias-Fernandez et al. (Biophys J, 112, 1176-1184, 2017), we have performed calculations using Caver 3.0 software with spherical probe of 0.8 Å radius, selected with a weighting coefficient of 1, clustering threshold of 12, shell radius of 18 Å, and shell depth of 4 Å.

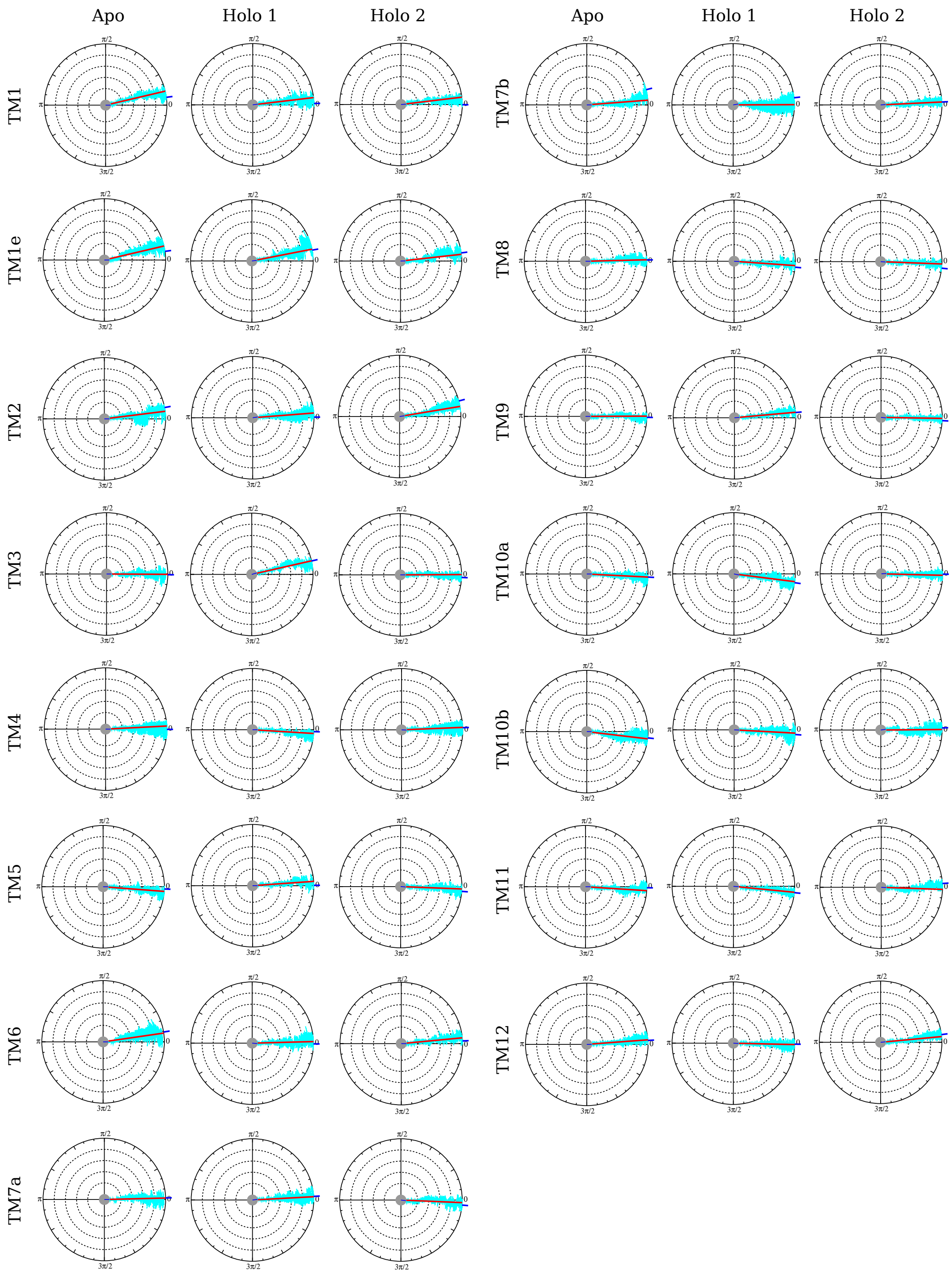

Figure S5. Rotation angle of the TM helices around the inertial axis during the MD simulations for GluT1 WT switching from the inward open conformation to the outward facing occluded state (Apo), and for the GluT1 dynamics in the presence of glucose in the outward facing (Holo 1) and inward open (Holo 2) conformations. The angle of rotation was measured in respect to the initial inward open structure. The blue ticks in the origin and on the side of the circle indicate the angle value for the first and last trajectory frames respectively, the red line corresponds to the mean angle value along the trajectory.
